# A Dewetting Model for Double-Emulsion Droplets

**DOI:** 10.3390/mi7110196

**Published:** 2016-11-01

**Authors:** Zhanxiao Kang, Pingan Zhu, Tiantian Kong, Liqiu Wang

**Affiliations:** 1 Department of Mechanical Engineering, The University of Hong Kong, Hong Kong, China; zxkang@connect.hku.hk (Z.K.); u3002025@connect.hku.hk (P.Z.); 2 Zhejiang Institute of Research and Innovation, The University of Hong Kong (HKU-ZIRI), Hangzhou 311300, Zhejiang, China; 3 Guangdong Key Laboratory for Biomedical Measurements and Ultrasound Imaging, Department of Biomedical Engineering, Shenzhen University, 3688 Nanhai Avenue, Shenzhen 518060, China; ttkong@szu.edu.cn

**Keywords:** double emulsion, dewetting force, equilibrium configuration, dewetting time

## Abstract

The evolution of double-emulsion droplets is of great importance for the application of microdroplets and microparticles. We study the driving force of the dewetting process, the equilibrium configuration and the dewetting time of double-emulsion droplets. Through energy analysis, we find that the equilibrium configuration of a partial engulfed droplet depends on a dimensionless interfacial tension determined by the three relevant interfacial tensions, and the engulfing part of the inner phase becomes larger as the volume of the outer phase increases. By introducing a dewetting boundary, the dewetting time can be calculated by balancing the driving force, caused by interfacial tensions, and the viscous force. Without considering the momentum change of the continuous phase, the dewetting time is an increasing function against the viscosity of the outer phase and the volume ratio between the outer phase and inner phase.

## 1. Introduction

**Figure 1:**
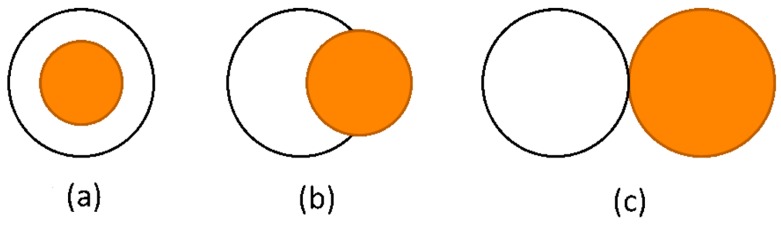
Morphologies of microdroplets: (**a**) Engulfing; (**b**) Partial-engulfing; (**c**) Non-engulfing.

Microdroplets have great potential in many applications. For example, they could be used for chemical reactions, therapeutic agent delivery and electronic paper [1,2]. Microdroplets used as a chemical reactor could enhance the intensity and uniformity of the reaction because of the tiny amount of reactant in the droplet. The structure of microdroplets usually plays an important role in these applications. For example, the core-shell structure (Figure 1a) can encapsulate and protect active ingredients and deliver them to the position with lesions [3,4,5,6]. Partial engulfing droplets (Figure 1b) are desirable for producing particles with two distinct sides such as the Janus particle which could be used as an emulsion stabilizer and building block of electronic paper [7]. The inner phase with active ingredients could also be released from the outer phase by the dewetting process, forming a non-engulfing structure [8] (Figure 1c). The structures of droplets are usually determined by the thermodynamic principle that droplets prefer the configuration with the lowest energy level [9,10]. Torza and Mason [9] studied droplet morphology in terms of spreading coefficients and obtained the theoretical relationship between the droplet morphology and spreading coefficients, which is used widely by many researchers [11,12,13,14,15,16,17]. In their study, the spreading coefficient was defined as $s_{i}=\sigma_{jk}-\left(\sigma_{ij}+\sigma_{ik}\right)$, where$\;\sigma_{ij},{\;\sigma }_{ik},\;\sigma_{jk}\;$denoted the three interfacial tensions between phases *i*, *j*, *k* correspondingly in a double-emulsion system. The droplet morphology could also be predicted directly by comparing interfacial tensions between different phases [17,18]. For the double emulsion, these studies predicted three droplet morphologies: engulfing, partial-engulfing and non-engulfing, shown in Figure 1, in the dewetting process where the outer phase and the inner phase separated from each other, forming a configuration with the minimum energy. However, the study on the interaction of the forces during the droplet dewetting process is very limited. As these forces are important for droplet configuration, especially when the driving force is small, kinetic factors such as viscosity may play the dominant role instead of the thermodynamic effect, making a thermodynamically non-engulfing droplet become partial-engulfing [8]. Furthermore, the droplet dewetting time is also determined by these forces, which is critical for the drug delivery process. Meanwhile, the precise prediction of the equilibrium configuration of droplets also depends heavily on these forces, which is vital for the precise fabrication of Janus particles. In this work, we prove that the interfacial tensions on the three-phase contact cycle are the exact forces driving the droplet dewetting process. Then, the equilibrium configuration is predicted by thermodynamic analysis. At last, the dewetting time of double emulsion is calculated. Hence, our study is of great importance for the fabrication and application of micro-emulsions by predicting the droplet configuration and dewetting time.

## 2. Driving Force of Dewetting Process

**Figure 2:**
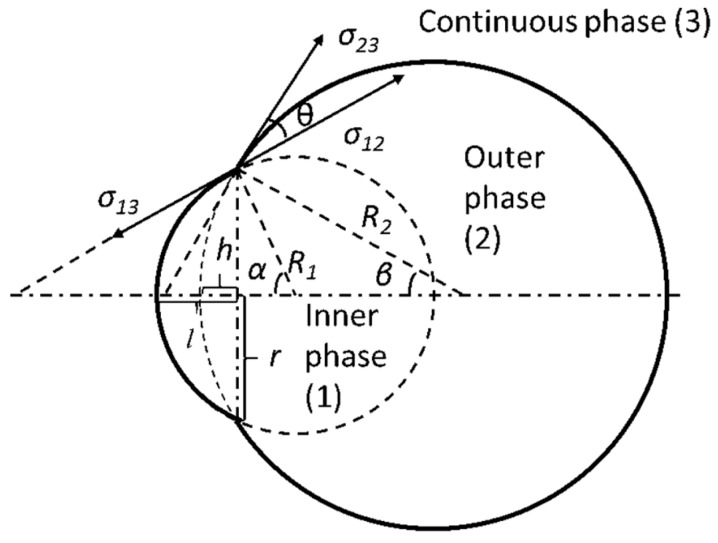
Schematic of the dewetting process of a double-emulsion droplet. Phases (1), (2) and (3) are inner, outer and continuous phases, respectively; σ*_ij_* is interfacial tension between phase *i* and *j*; *R*_1_ and *R*_2_ are the radii of inner and outer phases, respectively; *r* is the radius of a cycle formed by three-phase contact line; *l* is the length of phase (1) out of phase (2); *h* is the virtual height of phase (2) in phase (1).

The schematic of the dewetting process is shown in Figure 2. Phases (1), (2) and (3) are the inner, outer and continuous phases, respectively. Further, σ*_ij_* and *S_ij_* are the interfacial tension and interfacial area, respectively, between phase *i* and *j*, where *i,j =* 1,2,3. *R*_1_ and *R*_2_ are the radii of the inner phase and outer phase, respectively; *r* is the radius of a cycle which is formed by the three-phase contact line; *l* is the length of the inner phase out of the outer phase; *h* is the virtual height of the outer phase in the inner phase; α and β are the half central angles of the inner phase and outer phase with respect to the three-phase contact cycle; and θ is the angle between σ_12_ and σ_23_, shown in Figure 2. To simplify the analysis, we assume the morphology of the inner droplet remains unchanged during the entire dewetting process.

When the inner phase comes out a small distance such as dl from the outer phase, the angle α and β will correspondingly change dα and dβ, respectively. Thus, the work done by interfacial tension is

(1) $$\begin{array}{l} \delta W=(\sigma_{12}-\sigma_{13})\times 2\pi R_{1}\sin\alpha \times R_{1}d\alpha -\sigma_{23}\times 2\pi R_{2}\sin\beta \times R_{2}d\beta \\ \;=2\pi \left(\sigma_{12}-\sigma_{13}\right)R_{1}^{2}\sin\alpha d\alpha -2\pi \sigma_{23}R_{2}^{2}\sin\beta d\beta \end{array}$$

The minus sign in the second term is because of the decrease of $R_{2}$ in the whole dewetting process; thus, the work done by $\sigma_{23}$ is negative. The interfacial areas between the different phases are given by

(2) $$S_{12}=2\pi R_{1}^{2}\left(1+\cos\alpha \right)$$

(3) $$S_{13}=2\pi R_{1}^{2}\left(1-\cos\alpha \right)\;$$

(4) $$S_{23}=2\pi R_{2}^{2}\left(1-\cos\beta \right)$$

Hence, the variation of the Gibbs energy because of the change of the interfacial areas gives

(5) $$dG=\sigma_{12}dS_{12}+\sigma_{13}dS_{13}+\sigma_{23}dS_{23}=-2\pi \left(\sigma_{12}-\sigma_{13}\right)R_{1}^{2}\sin\alpha d\alpha +2{\pi \sigma }_{23}R_{2}^{2}\sin\beta d\beta$$

According to Equations (1) and (5), δW=−dG and we can thus conclude that the driving forces for the dewetting process of the double-emulsion droplet are the interfacial tensions along the three-phase contact cycle, a result consistent with the first law of thermodynamics. If the inner droplet deforms, the directions of $\sigma_{12}$ and $\sigma_{13}$ change, but no new force is induced. Hence, the three interfacial tensions remain the driving force of the dewetting process.

## 3. Equilibrium Configuration

The equilibrium configuration of a double-emulsion droplet is determined by its energy level, since droplets prefer the configuration with the lowest energy. In the following, the equilibrium configuration is derived through energy analysis.

According to mass conservation of the outer phase during the droplet dewetting process, we have

(6) $$\rho_{2}\frac{4}{3}\pi R_{21}^{3}=\rho_{2}\left[\frac{4}{3}\pi R_{2}^{3}-\frac{1}{3}\pi R_{1}^{3}{\left(L-2\right)}^{2}\left(L+1\right)-\frac{1}{3}\pi h^{2}\left(3R_{2}-h\right)\right]$$

where $R_{21}$ is the radius of the outer phase when the inner phase and outer phase separate completely, $\rho_{2}$ is the density of the outer phase and $L=l/R_{1}$ which is a dimensionless position used to denote the droplet configuration in this section. Thus, the inner phase is totally in the outer phase when L=0, and they separate completely from each other when L=2. Let $k=R_{21}/R_{1}$ characterizing the volume ratio between the outer phase and inner phase, and then we have $h^{2}/R_{2}^{2}\;\sim \;h^{2}/R_{1}^{2}\;\sim \;O\left(10^{-2}\right)$ when *k* ≥ 1 (O is a sign denoting the order of magnitude), so the last term of Equation (6) can be ignored.

Assuming the density of the outer phase to be constant, we can get

(7) $$\frac{4}{3}\pi R_{21}^{3}=\frac{4}{3}\pi R_{2}^{3}-\frac{1}{3}\pi R_{1}^{3}{\left(L-2\right)}^{2}\left(L+1\right)$$

Hence, Equation (7) could be rearranged to

(8) $$R_{2}=KR_{1}$$

where $K=1/2\times {\left(8k^{3}+8-6L^{2}+2L^{3}\right)}^{1/3}$, which could be regarded as a shape factor characterizing the shape change of the outer phase with respect to *L* and *k*.

Therefore, the total Gibbs energy of the system is given by, based on geometry analysis,

(9) $$G=\sigma_{13}S_{13}+\sigma_{12}S_{12}+\sigma_{23}S_{23}=2\pi R_{1}^{2}\sigma_{12}\left[\left(2-L\right)+xL+y\left(K^{2}+K\sqrt{K^{2}-2L+L^{2}}\right)\right]$$

where $x=\sigma_{13}/\sigma_{12},\;y=\sigma_{23}/\sigma_{12}$. Furthermore, we have the dimensionless Gibbs energy normalized by the Gibbs energy of the inner phase:

(10) $$G^{*}=\frac{G}{4\pi R_{1}^{2}\sigma_{12}}=\frac{1}{2}\left[\left(2-L\right)+xL+y\left(K^{2}+K\sqrt{K^{2}-2L+L^{2}}\right)\right]$$

Taking the derivative of the dimensionless Gibbs energy (Equation (10)) with respect to *L*, we have

(11) $$\frac{dG^{*}}{dL}=-\frac{1}{2}+\frac{1}{2}x+\frac{1}{4}y\left[\frac{L^{2}-2L}{K}+\frac{L^{2}-2L+4KL-4K}{2\sqrt{K^{2}-2L+L^{2}}}+\frac{\left(L^{2}-2L\right)\sqrt{K^{2}-2L+L^{2}}}{2K^{2}}\right]$$

By solving Equation (11) = 0, the equilibrium configuration could be described implicitly by

(12) $$\frac{1}{2}\left[\frac{L^{2}-2L}{K}+\frac{L^{2}-2L+4KL-4K}{2\sqrt{K^{2}-2L+L^{2}}}+\frac{\left(L^{2}-2L\right)\sqrt{K^{2}-2L+L^{2}}}{2K^{2}}\right]=\Sigma$$

where $\Sigma =\left(\sigma_{12}-\sigma_{13}\right)/\sigma_{23}$, which is a dimensionless interfacial tension determined by the three interfacial tensions in the double-emulsion system.

Therefore, the equilibrium configuration denoted by *L* is determined by the dimensionless interfacial tension Σ for a given radius ratio *k*. Table 1 gives the interfacial tensions between poly(2-phenylpropylme-thylsiloxane) (PPPMS), poly(octylmethylsiloxane) (POMS), poly(3,3,3-trifluoropropylmethylsiloxane) (PFPMS) and water (the surfactant concentration of sodium dodecyl sulfate (SDS) is 5 mM) [15], which will be applied to analyze the equilibrium configuration of a double-emulsion droplet.

**Figure 3:**
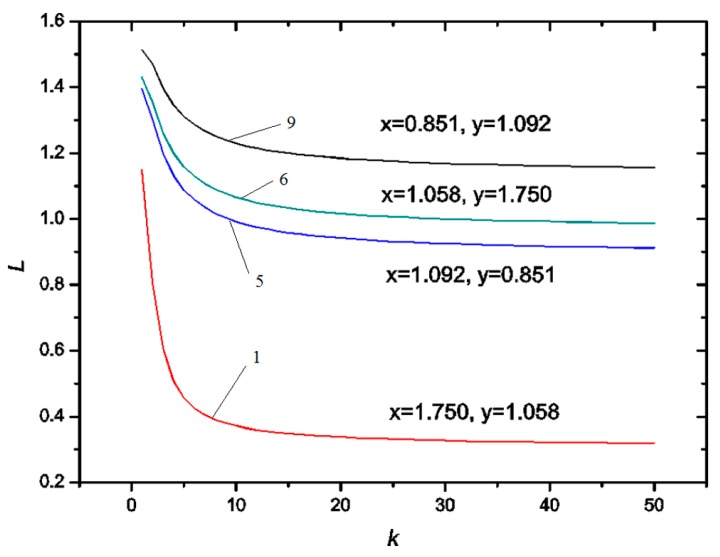
Variation of equilibrium position *L* (*L* = *l*/*R*_1_) with respect to radius ratio *k* (*k* = *R*_21_/*R*_1_).

Figure 3 shows the variation of the equilibrium position *L* (*L* = *l*/*R*_1_) with respect to the radius ratio (*k* = *R*_21_/*R*_1_) for systems 1, 5, 6 and 9 in Table 1. It indicates that the equilibrium position *L* decreases dramatically with the increase of *k* when *k* is small such as *k* < 10, but gradually tends to be a constant when *k* is large enough. Therefore, the inner phase tends to be engulfed into the outer phase as the volume of the outer phase increases. However, if the volume of the outer phase is large enough, the volume of the inner phase engulfed in the outer phase tends to be constant. Furthermore, comparing the data between system 1 and system 9, Figure 3 denotes the equilibrium position *L* decreases with the increase of *x* (σ_13_/σ_12_). On the other hand, the data of system 5 and system 6 shows that the equilibrium position *L* increases with the increase of *y* (σ_23_/σ_12_).

**Figure 4:**
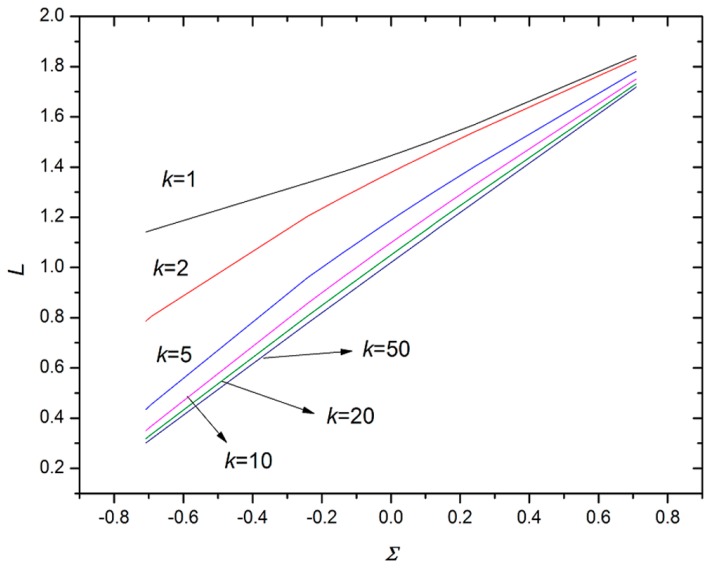
Variation of equilibrium position *L* (*L* = *l*/*R*_1_) with respect to dimensionless interfacial tension Σ (Σ = (σ_12_ − σ_13_)/σ_23_).

Figure 4 demonstrates the variation of equilibrium position *L* with respect to dimensionless interfacial tension Σ ((σ_12_ − σ_13_)/σ_23_), which indicates that the equilibrium position *L* linearly depends on Σ approximately, providing a practical approach to predict the droplet configuration. The equilibrium position *L* increases with the increase of Σ, which means the larger the Σ, the smaller the part of the inner phase engulfed in the outer phase (this qualitatively agrees with the results in the literature [18]). Furthermore, with the increase of the volume ratio *k* (*k* = *R*_21_/*R*_1_), the slope of the *L* variation with respect to Σ increases, which means the equilibrium position is more sensitive to the dimensionless interfacial tension at a larger volume ratio of the outer to the inner phase. However, the slope tends to a constant when *k* is sufficiently large, indicating the existence of a saturated equilibrium position at a large volume ratio, which agrees with the results in Figure 3. At the saturated condition, the equilibrium position is determined by the dimensionless interfacial tension with negligible influence of the volume ratio.

## 4. Dewetting Time of Double-Emulsion Droplet

As the velocities of the inner phase and outer phase are usually very small in the dewetting process, the droplets’ movement relative to each other could be considered approximately as Stokes flow; the resistance force per unit area is thus f=3ηu/2R , where η is the dynamic viscosity of the surrounding fluid and u is the relative velocity of the adjacent phases.

Assuming the viscosity of the continuous phase is sufficiently small, the momentum change of the continuous phase is neglected. Based on the momentum conservation of the inner phase and outer phase, we have

(13) $$m_{1}u_{1}=m_{2}u_{2}$$

where $u_{1}$ and $u_{2}$ are the velocities of the inner and outer phases during the dewetting process, respectively, which depend on the dimensionless position *L*.

The driving force component in droplets separating direction is given by

(14) $$F_{R}=\left[\left(\sigma_{12}-\sigma_{13}\right)\sin\alpha +\sigma_{23}\sin\beta \right]\times 2\pi r=2{\pi \sigma }_{12}R_{1}L\left(2-L\right)\left(1-x+\frac{y}{K}\right)$$

With a sufficiently small viscosity of the continuous phase, the viscous resistance force generated by the outer phase is dominant and is thus given by, based on Stokes flow,

(15) $$F_{D}=\frac{3\eta_{2}\left(u_{1}+u_{2}\right)}{2R_{1}}S_{12}=3{\pi \eta }_{2}u_{1}R_{1}\left(1+\frac{1}{Ak^{3}}\right)\left(2-L\right)$$

where $A=\rho_{2}/\rho_{1},\;$in which $\rho_{1}$ and $\rho_{2}$ are the density of the inner phase and outer phase, respectively, and${\;\eta }_{2}$ is the dynamic viscosity of the outer phase. According to energy conservation, we have

(16) $$\int_{0}^{L}\left(F_{R}-F_{D}\right)dL=\frac{1}{2}m_{1}u_{1}^{2}+\frac{1}{2}m_{2}u_{2}^{2}$$

Therefore, the dewetting time could be calculated by

(17) $$t=\int_{0}^{L_{eq}}\frac{1}{u_{1}+u_{2}}dL$$

where $L_{eq}$ is the value of L in the equilibrium configuration of the double emulsion.

Solving Equations (13)–(17) yields the dewetting time. However, in the initial stage where L is small, the driving force is too small, which leads to an extremely long dewetting time that is not consistent with experimental results; thus, a dewetting boundary should be introduced here. It is better to define the dewetting boundary by experiment. According to Einstein’s theory, the average dimensionless displacement per unit time normalized by the radius of the inner phase, induced by Brownian motion, is on the order of $O\left(10^{-3}\right)$ for an emulsion with a diameter of 100 μm at 300 K in water. Hence, to get the solution of this problem, we define 0.5% of the diameter of the inner phase as the dewetting boundary, which means *L* = 0.01, so the dewetting time could be calculated by

(18) $$t=\int_{0.01}^{L_{eq}}\frac{1}{u_{1}+u_{2}}dL$$

**Figure 5:**
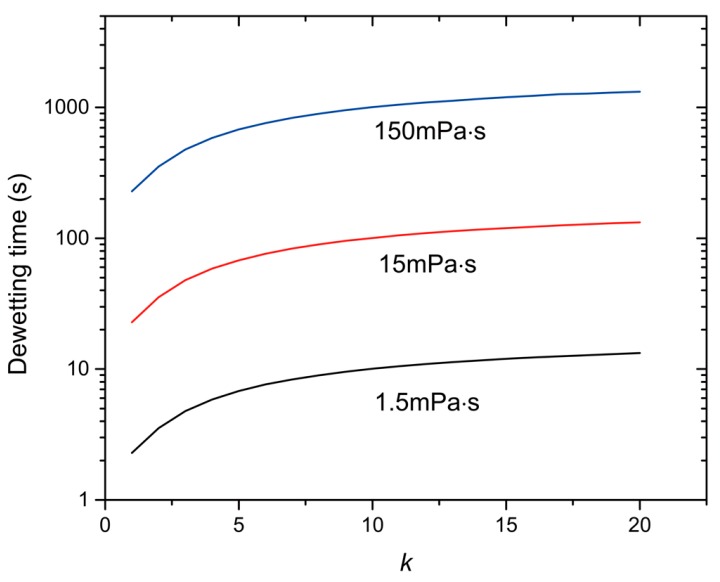
Dewetting time of double emulsion for the poly(2-phenylpropylme-thylsiloxane)/poly(3,3,3-trifluoropropylmethylsiloxane)/water (PPPMS/PFPMS/water) system under different viscosities of the outer phase.

Figure 5 shows the dewetting time from the numerical calculation for the PPPMS/PFPMS/water system (*R*_1_ = 0.1 mm, *ρ*_1_ = 1.02 g/mL, η_2_ = 1.5/15/150 mPa·s, σ_12_ = 15.4 mN/m, *A =* 1.25, *x* = 0.916, *y* = 0.779). From Figure 5, we see that the dewetting time increases with the increase of volume ratio *k* and the viscosity of the outer phase. According to momentum conservation, with the increase of volume ratio *k*, a larger outer phase associates with a smaller relative velocity, and thus a longer dewetting time. Based on Equations (16) and (17), the dewetting time is determined by the balance of the driving force of interfacial tensions, and the viscous force. Assuming a constant driving force, a more viscous outer phase leads to a smaller velocity based on Equation (15). As such, the dewetting time becomes longer (qualitatively agrees with the results in the literature [8]). The orders of the dewetting time are, respectively, O(1 s), O(10 s) and O(100 s), while the orders of the viscosity of the outer phase are O(0.001 Pa·s), O(0.01 Pa·s) and O(0.1 Pa·s), respectively. On the other hand, if the viscosity of the continuous phase is so large, the momentum change of the continuous phase cannot be ignored and, thus, momentum conservation, Equation (13), is not satisfied. As a result, the momentum of the inner and outer phase becomes smaller, leading to a longer dewetting time.

## 5. Concluding Remarks

We analyze the dewetting process of a double emulsion analytically based on force analysis. The equilibrium configuration of double-emulsion droplets depends on a dimensionless interfacial tension determined by the three interfacial tensions in the system. The outer phase engulfs more inner phase when the volume ratio between the outer phase and inner phase is larger, but the engulfing part of the inner phase is almost constant at a sufficiently large volume ratio. By balancing the interfacial tensions and viscous force in the dewetting process, with an introduced dewetting boundary, the dewetting time of double-emulsion droplets can be calculated based on momentum and energy conservation with a sufficiently small viscosity of the continuous phase. A large volume ratio between the outer phase and inner phase leads to the increase of the dewetting time based on momentum conservation. Meanwhile, the larger the viscosity of the outer phase, the longer the dewetting time required. As the precise configuration and the dewetting time of double-emulsion droplets could be calculated, it is of great importance for the application of double emulsions such as in drug delivery and electronic displays.

## Figures and Tables

**Table 1:** Interfacial tension (mN/m). PPPMS: poly(2-phenylpropylme-thylsiloxane); POMS: poly(octylmethylsiloxane); PFPMS: poly(3,3,3-trifluoropropylmethylsiloxane).

NO.	Phase (1)	Phase (2)	Phase (3)	$\sigma_{12}$	$\sigma_{13}$	$\sigma_{23}$	Σ	*x* ($\frac{\sigma_{13}}{\sigma_{12}}$)	*y*($\frac{\sigma_{23}}{\sigma_{12}}$)
1	PFPMS	water	POMS	12	21	12.7	−0.7087	1.750	1.058
2	POMS	water	PFPMS	12.7	21	12	−0.6917	1.654	0.945
3	PFPMS	water	PPPMS	12	15.4	14.1	−0.2411	1.283	1.175
4	water	PFPMS	PPPMS	12	14.1	15.4	−0.1364	1.175	1.283
5	PPPMS	water	PFPMS	14.1	15.4	12	−0.1083	1.092	0.851
6	water	PFPMS	POMS	12	12.7	21	−0.0333	1.058	1.750
7	water	POMS	PFPMS	12.7	12	21	0.0333	0.945	1.654
8	PPPMS	PFPMS	water	15.4	14.1	12	0.1083	0.916	0.779
9	water	PPPMS	PFPMS	14.1	12	15.4	0.1364	0.851	1.092
10	PFPMS	PPPMS	water	15.4	12	14.1	0.2411	0.779	0.916
11	POMS	PFPMS	water	21	12.7	12	0.6917	0.605	0.571
12	PFPMS	POMS	water	21	12	12.7	0.7087	0.571	0.605
